# Supplementary material for: The essential role of YAP O-GlcNAcylation in high-glucose-stimulated liver tumorigenesis
Source: Nat Commun. 2017 May 5;8:15280. doi: 10.1038/ncomms15280 (PMC5424161; doi:10.1038/ncomms15280)
Supplement: Supplementary Information — Supplementary Figures and Supplementary Table [file ncomms15280-s1.pdf]

a

YAP	CTGF +	CTGF ++	CTGF +++	Total
+	54	22	11	87
++	17	35	16	68
+++	8	13	32	53
Total	79	70	59	208
		x^2 test,		p =0.000

YAP	Ki67 +	Ki67 ++	Ki67 +++	Total
+	42	31	14	87
++	10	35	23	68
+++	5	14	34	53
Total	57	80	71	208
		x^2 test,		p =0.000

YAP	TEAD +	TEAD ++	TEAD +++	Total
+	27	31	29	87
++	27	26	15	68
+++	24	15	14	53
Total	78	72	58	208
		x^2 test,		p =0.331

O-GlcNAc	CTGF +	CTGF ++	CTGF +++	Total
+	36	13	5	54
++	26	35	13	74
+++	17	22	41	80
Total	79	70	59	208
		x^2 test,		p =0.000

O-GlcNAc	Ki67 +	Ki67 ++	Ki67 +++	Total
+	41	11	2	54
++	12	42	20	74
+++	4	27	49	80
Total	57	80	71	208
		x^2 test,		p =0.000

O-GlcNAc	TEAD +	TEAD ++	TEAD +++	Total
+	26	14	14	54
++	26	24	24	74
+++	26	34	20	80
Total	78	72	58	208
		x^2 test,		p =0.210

b

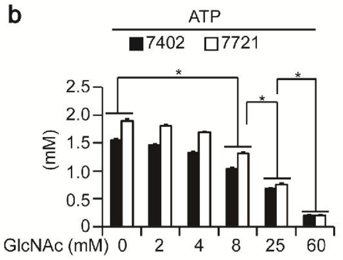

c

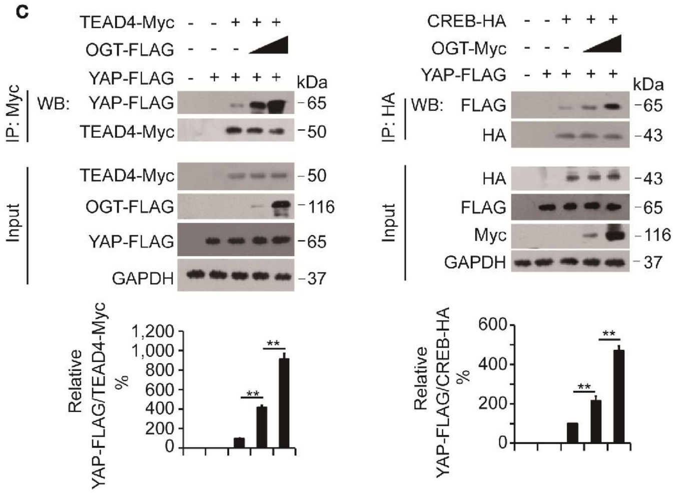

Supplementary Figure 1. Analysis of the TMA, reduction of intracellular ATP by GlcNAc and induction of YAP binding activities by OGT.

(a) Analysis of TMA. Data from TMA in Fig. 1a were analyzed by a chi-square test.

(b) GlcNAc reduced ATP at high concentrations. Intracellular ATP from cell lysates (1.25×10^6 cells/100 μl lysis buffer) was examined by an ATP testing kit from Beyotime (Haimen, China) in Bel-7402 and SMMC-7721 cells treated with increasing concentrations of GlcNAc as indicated.

(c) OGT facilitated the binding between YAP and TEAD or CREB. In OGT-FLAG or OGT-Myc and YAP-FLAG transfected Bel-7402 cells, TEAD-Myc and CREB-HA was immunoprecipitated with anti-Myc (left) and anti-HA (right) antibodies respectively, followed by Western blot using an anti-FLAG antibody. The relative ratios between YAP-FLAG and TEAD4-Myc or CREB-HA were also shown, and the data from cells co-transfected with YAP-FLAG and TEAD4-Myc or CREB-HA without OGT overexpression are arbitrarily set to 100%.

Representative images are shown, and the data are expressed as the means + SD from three independent experiments. *, $p < 0.05$ and **, $p < 0.01$ indicate statistical significance. The data from Supplementary Fig. 1b-c were analyzed by one-way ANOVA test.

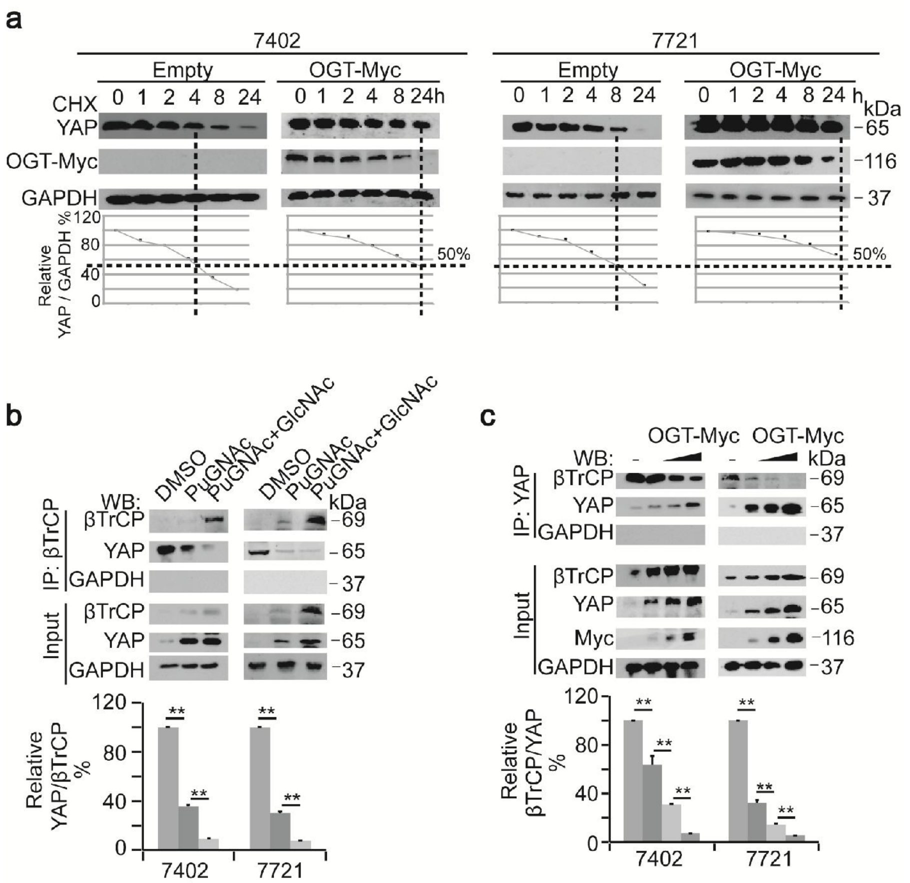

a
7402
7721
Empty
OGT-Myc
CHX
0 1 2 4 8 24h
0 1 2 4 8 24 h
kDa
YAP
OGT-Myc
GAPDH
65
116
37
Relative YAP / GAPDH %
120
80
40
0
50%
b
WB:
DMSO
PuGNAc
PuGNAc+GlcNAc
IP: βTrCP
Input
βTrCP
YAP
GAPDH
69
Relative YAP/βTrCP %
**
c
OGT-Myc
IP: YAP
Myc
Relative βTrCP/YAP %

Supplementary Figure 2. OGT prolongs half-life of YAP through preventing βTrcP interact with YAP.

(a) OGT overexpression increased YAP stability. Protein synthesis was blocked by treatment with CHX (50 μg/ml) for the indicated times. The half-life of endogenous YAP in control and Bel-7402 or SMMC-7721 cells with OGT overexpressed, as measured by Western blot analysis. The levels of YAP were normalized to those of GAPDH, and the 0 h points are arbitrarily set to 100%.

(b) Stimulation of O-GlcNAcylation blocked the interaction between βTrCP and YAP. Bel-7402 and SMMC-7721 cells were treated with DMSO, PuGNAc with or without GlcNAc for 24 h. βTrCP was immunoprecipitated by anti-βTrCP antibodies, and the associations of YAP were detected by anti-YAP antibodies. The levels of YAP were normalized to those of βTrCP in the IP samples, and the data from cells treated with DMSO are arbitrarily set to 100%.

(c) OGT overexpression blocked the interaction between βTrCP and YAP. Bel-7402 and SMMC-7721 cells were transfected with or without increasing concentrations of OGT expressing plasmids (1–3 μg/well of 6-well plate). YAP was immunoprecipitated by anti-YAP antibodies, and the associations of βTrCP were detected by anti-βTrCP antibodies. The levels of βTrCP were normalized to those of YAP in the IP samples, and the data from cells without treatments are arbitrarily set to 100%.

Representative images are shown, and the data are expressed as the means + SD from three independent experiments. **, $p < 0.01$ indicates statistical significance. The data from Supplementary Fig. 2b-c were analyzed by one-way ANOVA test.

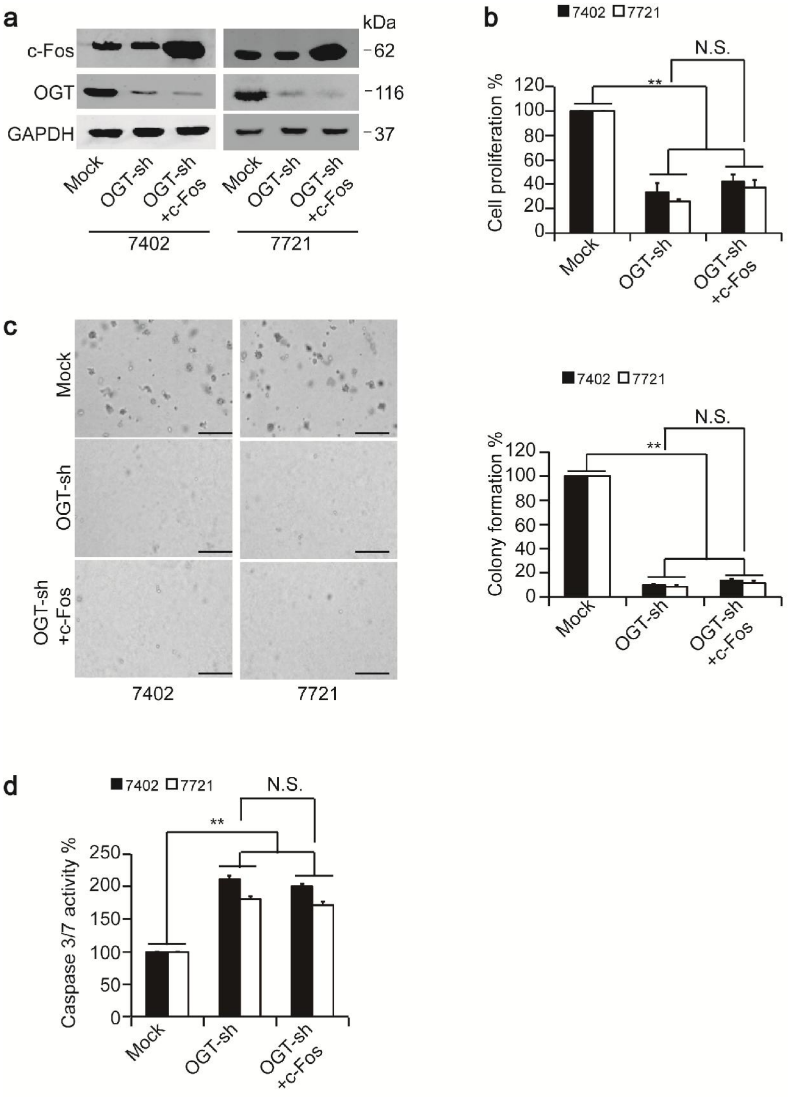

Supplementary Figure 3. Impaired transformative phenotypes by knocking down OGT cannot be reversed by c-Fos.

(a) Representative Western blotting images of c-Fos and OGT in control cells, Bel-7402 and SMMC-7721 cells with OGT knocked down with or without simultaneous overexpression of c-Fos.

(b-d) Impaired transformative phenotypes induced by knockdown of OGT could not be affected by overexpression of c-Fos. Cell proliferation, colony formation capacity and Caspase 3/7 activities were measured by an MTT-based assay (b), soft agar colony formation assay (c) and Caspase 3/7 Glo luciferase assay (d) respectively in Bel-7402 and SMMC-7721 cells under different treatments as indicated. Scale bar, 500 μm.

The data are shown as the means + SD from three independent experiments. **, $p < 0.01$ indicates statistical significance. N.S., not significant. The data from Supplementary Fig. 3b-d were analyzed by a one-way ANOVA test. The data from Mock group are arbitrarily set to 100%.

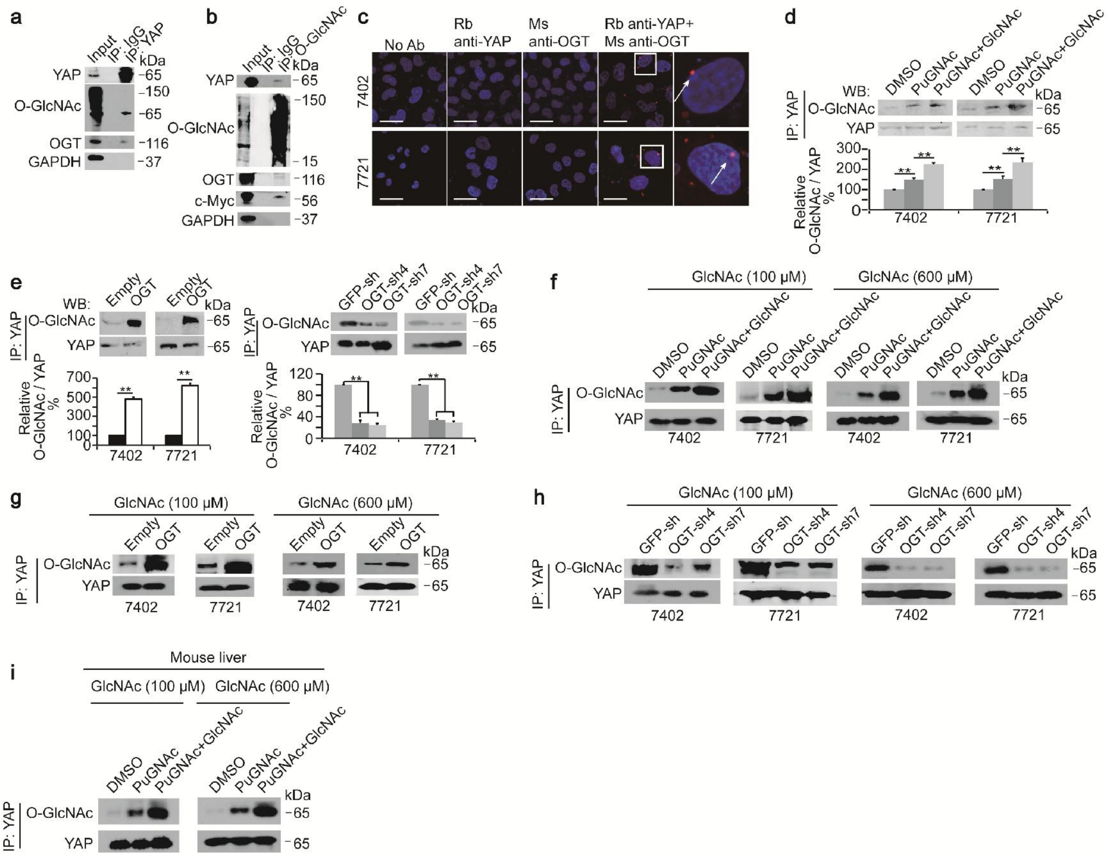

a
Input
IP: IgG
IP: YAP
kDa
YAP
O-GlcNAc
OGT
GAPDH
b
Input
IP: IgG
IP: O-GlcNAc
c
No Ab
Rb anti-YAP
Ms anti-OGT
Rb anti-YAP+ Ms anti-OGT
7402
7721
c-Myc
d
WB:
DMSO
PuGNAc
PuGNAc+GlcNAc
IP: YAP
Relative O-GlcNAc / YAP %
e
Empty
OGT
GFP-sh
OGT-sh4
OGT-sh7
f
GlcNAc (100 μM)
GlcNAc (600 μM)
g
h
i
Mouse liver

Supplementary Figure 4. O-GlcNAcylation of YAP

(a-b) Interactions among YAP, O-GlcNAc and OGT. Endogenous YAP (a) and O-GlcNAc (b) were immunoprecipitated by anti-YAP and anti-O-GlcNAc antibodies respectively. Co-immunoprecipitation of OGT and O-GlcNAc (a) or YAP, OGT and c-Myc (b) were analyzed by western blotting using indicated antibodies in Bel-7402 cells.

(c) The interactions between endogenous YAP and OGT in Bel-7402 and SMMC-7721 cells were measured by a Duolink™ proximity ligation (PLA) kit using anti-YAP and anti-OGT antibodies, as indicated. Parallel negative controls were also analyzed by using indicated antibodies. The areas with red signals were indicated and enlarged by a square. Scale bar, 50 μm. The arrows show strong interaction signals. Ms, mouse origin; Rb, rabbit origin.

(d-e) PuGNAc, GlcNAc and OGT promoted YAP O-GlcNAcylation. Bel-7402 and SMMC-7721 cells were treated with DMSO, PuGNAc (25 μM) with or without GlcNAc (4 mM) (d), transfected with OGT-expressing plasmid or infected with OGT-sh (e). Endogenous YAP was immunoprecipitated by an anti-YAP antibody. Co-immunoprecipitation of O-GlcNAc was analyzed using an anti-O-GlcNAc antibody. The O-GlcNAc levels of YAP were normalized to the YAP, and the data from the control group (treated with either DMSO, empty vectors or GFP-sh) are arbitrarily set to 100%.

(f-h) Detection of O-GlcNAcylation of YAP by RL2 antibodies in cells maintained under low or high GlcNAc concentrations. PuGNAc, GlcNAc and OGT promoted YAP O-GlcNAcylation in human liver cancer cells. Bel-7402 and SMMC-7721 cells were maintained under either low (100 μM) or high concentrations (600 μM) of GlcNAc before and during treating with DMSO, PuGNAc (25 μM) with or without additional GlcNAc (4 mM) (f), infected with OGT-expressing plasmid (g) or indicated OGT-sh (h). Endogenous YAP was immunoprecipitated by an anti-YAP antibody. Co-immunoprecipitation of O-GlcNAc was analyzed using an anti-O-GlcNAc antibody (RL2).

(i) PuGNAc and GlcNAc stimulated O-GlcNAcylation of YAP in mouse liver. Primary hepatocytes that obtained from 5-week Balb/c male mice were cultured in either 100 or 600 μM GlcNAc for 24 h before treating with DMSO, PuGNAc (25 μM) with or without additional GlcNAc (4 mM). Endogenous YAP was immunoprecipitated by an anti-YAP antibody. Co-immunoprecipitation of O-GlcNAc was analyzed using an anti-O-GlcNAc antibody (RL2). Representative images are shown, and the data are expressed as the means + SD from three independent experiments. **, $p < 0.01$ indicates statistical significance. The data from Supplementary Fig. 4d and 4e (right panel) were analyzed by a one-way ANOVA test. The

data from Supplementary Fig. 4e (left panel) were analyzed by a Student’s t-test.

a

YAP1_HUMAN 1496 Transcriptional coactivator YAP1 OS=Homo sapiens GN=YAP1 PE=1 SV=2

	Score	Mass	Matches	Sequences	emPAI	
YAP1_HUMAN	1496	34484	64(51)	11(11)	1.71	Transcriptional coactivator YAP1 OS Homo sapiens GN YAP1 PE 1 SV 2

▼64 peptide matches (17 non-duplicate, 47 duplicate)

Query	Dupes	Observed	Mr(expt)	Mr(calc)	ppm	M	Score	Expect	Rank	U	Peptide
3573	▶6	626.3375	1250.6605	1250.6547	4.65	1	46	0.00033	▶1	U	K.LDKESFLTWL.-
5689	▶7	510.6189	1528.8350	1528.8289	3.93	1	28	0.04	▶1	U	R.KLPDSFFKPPEPK.S
7396	▶2	909.3950	1816.7755	1816.7785	-1.67	0	115	1.9e-011	▶1	U	R.DESTDSGLSMSSYSVPR.T
8075	▶3	975.9518	1949.8886	1949.9040	-7.89	0	87	7.3e-009	▶1	U	R.GDSETDLEALFNAVMNPK.T
8077	▶3	650.9723	1949.8950	1949.9040	-4.61	0	73	9.9e-007	▶1	U	R.GDSETDLEALFNAVMNPK.T
8528	▶1	1017.9844	2033.9543	2033.9595	-2.57	0	65	7.2e-006	▶1	U	R.YFLNHIDQTTTWQDPR.K
8532	▶14	678.9939	2033.9599	2033.9595	0.15	0	89	2e-007	▶1	U	R.YFLNHIDQTTTWQDPR.K
9569		1109.0195	2216.0245	2216.0460	-9.68	0	53	0.00025	▶1	U	R.QSSFEIPDDVPLPAGWEMAK.T
9578		740.0217	2217.0432	2216.0460	450	0	18	0.029	▶1	U	R.QSSFEIPDDVPLPAGWEMAK.T
11274		919.4445	2755.3115	2755.3083	1.18	0	22	0.038	▶1	U	R.SQLPTLEQDGGTQNPVSSPGMSQELR.T
11265	▶2	924.7741	2771.3005	2771.3032	-0.98	0	65	4e-006	▶1	U	R.SQLPTLEQDGGTQNPVSSPGMSQELR.T + Oxidation (M)
12683	▶2	1080.1454	3237.4143	3237.4368	-6.94	0	34	0.0048	▶1	U	R.TPDDFLNSVDEMDTGDTINQSTLPSQQNR.F
13311	▶4	884.4688	3533.8459	3533.8591	-3.73	0	91	8.6e-009	▶1	U	R.AHSSPASLQLGAVSPGTLTPTGVVSGPAAIPIAQHLR.Q
13314	▶2	707.7769	3533.8479	3533.8591	-3.16	0	40	0.00018	▶1	U	R.AHSSPASLQLGAVSPGTLTPTGVVSGPAAIPIAQHLR.Q
13653	▼1	1113.5046	5562.4863	5560.4513	366	0	39	0.0012	▶1	U	K.AMLSQMNVTAPTSPPVQQNMMNSASGPLPDGWEQAMTQDGEIYYINHK.N + GLCNAC (ST); 5 Oxidation (M)
	13654	1113.5084	5562.4863	5560.4513	369	0	(33)	0.0033	▶1	U	K.AMLSQMNVTAPTSPPVQQNMMNSASGPLPDGWEQAMTQDGEIYYINHK.N + GLCNAC (ST); 5 Oxidation (M)
13663		1397.4406	5585.7331	5585.7690	-6.43	0	18	0.02	▶1	U	M.DPGQQPPPQPAPQGQGQPPSQPPQGQGPPSGPGQPAPAATQAAPQAPPAGHQIVHVR.G
13664		1118.1553	5585.7400	5585.7690	-5.19	0	16	0.03	▶1	U	M.DPGQQPPPQPAPQGQGQPPSQPPQGQGPPSGPGQPAPAATQAAPQAPPAGHQIVHVR.G

Supplementary Figure 5. Mascot search results for mass spectrum.

(a) Mascot search result for Nano-LC-QTOF-MS analysis of an in-gel YAP trypsin digest. By using the Swiss-Prot database, O-GlcNAcylation of YAP at Thr241 was identified.

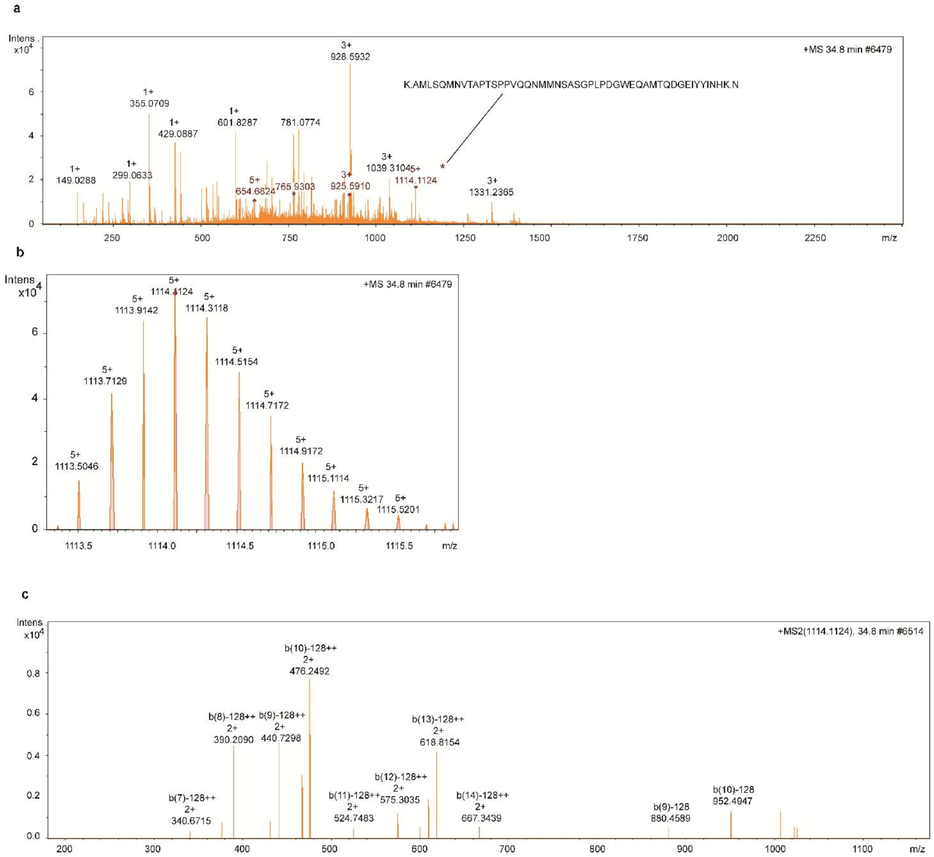

Supplementary Figure 6. The O-GlcNAcylated YAP peptide (#13653) in mass spectrum.

(a) Mas spectrum of in-gel YAP trypsin digest. m/z 0-2500, 34.8 min. The O-GlcNAcylated peptide is indicated by an asterisks (*).

(b) The isotope peak of O-GlcNAcylated peptide #13653 in mass spectrum. m/z 1113.50-1115.75, 34.8 min.

(c) Mass pectrum of O-GlcNAcylated peptide #13653. m/z 200-1160, 34.8min. The Neutral loss of each Oxidation is -64.

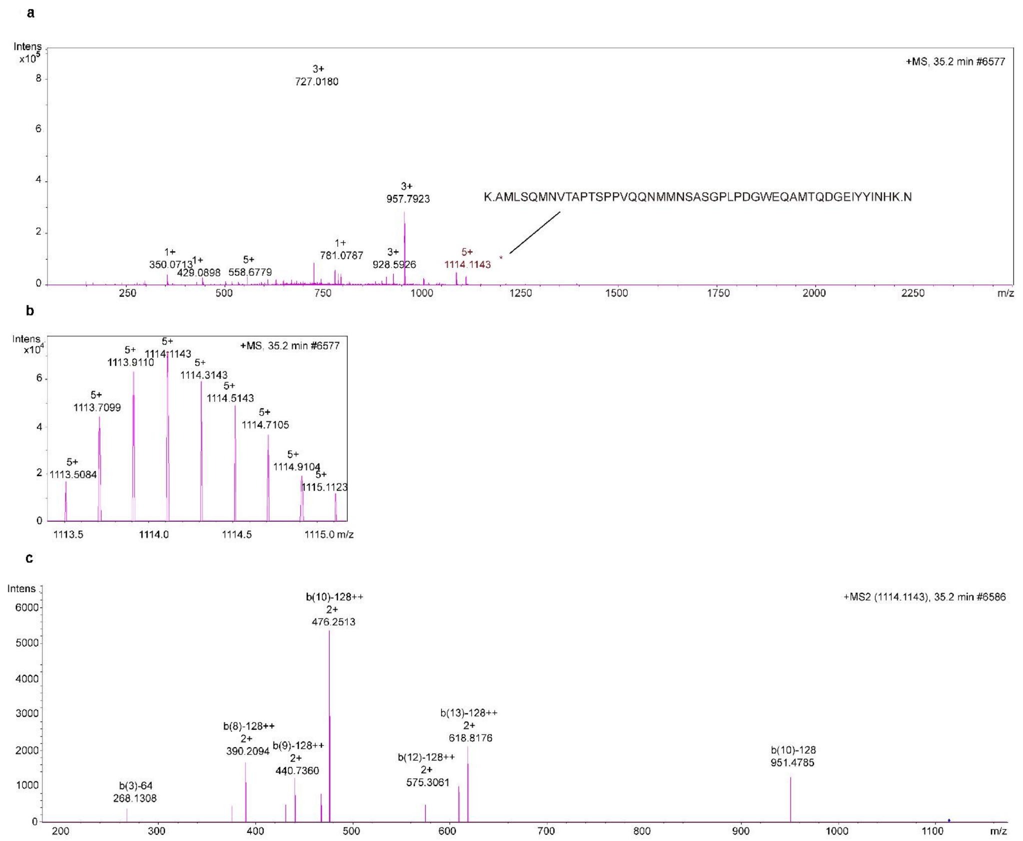

Supplementary Figure 7. The O-GlcNAcylated YAP peptide (#13654) in mass spectrum.

(a) Mas spectrum of in-gel YAP trypsin digest. m/z 0-2500, 35.2 min. The O-GlcNAcylated peptide is indicated by an asterisks (*).

(b) The isotope peak of O-GlcNAcylated peptide #13654 in mass spectrum. m/z 1113.5-1115.2, 35.2 min.

(c) Mass pectrum of O-GlcNAcylated peptide #13654. m/z 200-1160, 35.2 min. The Neutral loss of each Oxidation is -64.

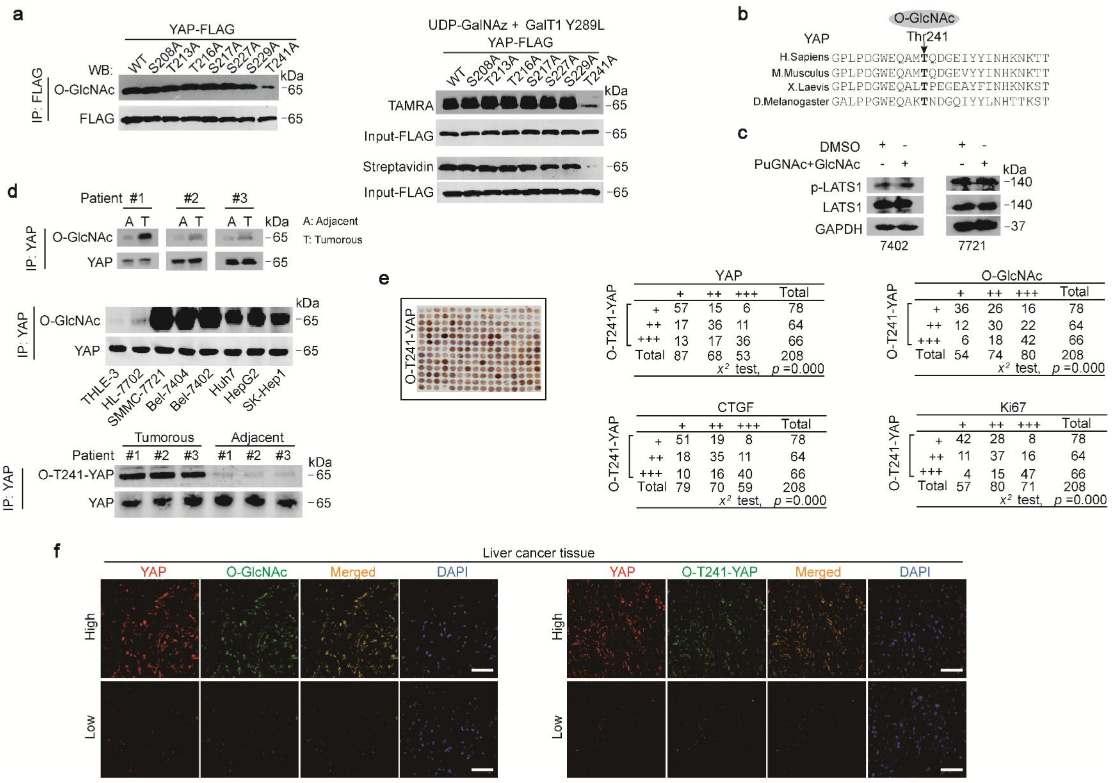
a
YAP-FLAG
WB: WT S208A T213A T216A S217A S227A S229A T241A
kDa
IP: FLAG
O-GlcNAc -65
FLAG -65
UDP-GalNAz + GalT1 Y289L
YAP-FLAG
WT S208A T213A T216A S217A S227A S229A T241A
kDa
TAMRA -65
Input-FLAG -65
Streptavidin -65
Input-FLAG -65
b
O-GlcNAc
YAP
Thr241
H.Sapiens GPLPDGWEQAMTQDGEIYYINHKNKTT
M.Musculus GPLPDGWEQAMTQDGEVYYINHKNKTT
X.Laevis GPLPDGWEQALTPEGEAYFINHKNKST
D.Melanogaster GALPPGWEQAKTNDGQIYYLNHTTKST
c
DMSO + - + -
PuGNAc+GlcNAc - + - +
kDa
p-LATS1 -140
LATS1 -140
GAPDH -37
7402 7721
d
Patient #1 #2 #3
A T A T A T
kDa
A: Adjacent
T: Tumorous
IP: YAP
O-GlcNAc -65
YAP -65
kDa
IP: YAP
O-GlcNAc -65
YAP -65
THLE-3 HL-7702 SMMC-7721 Bel-7404 Bel-7402 Huh7 HepG2 SK-Hep1
Tumorous Adjacent
Patient #1 #2 #3 #1 #2 #3
kDa
IP: YAP
O-T241-YAP -65
YAP -65
e
O-T241-YAP
YAP
O-T241-YAP + ++ +++ Total
+ 57 15 6 78
++ 17 36 11 64
+++ 13 17 36 66
Total 87 68 53 208
x² test, p =0.000
O-GlcNAc
O-T241-YAP + ++ +++ Total
+ 36 26 16 78
++ 12 30 22 64
+++ 6 18 42 66
Total 54 74 80 208
x² test, p =0.000
CTGF
O-T241-YAP + ++ +++ Total
+ 51 19 8 78
++ 18 35 11 64
+++ 10 16 40 66
Total 79 70 59 208
x² test, p =0.000
Ki67
O-T241-YAP + ++ +++ Total
+ 42 28 8 78
++ 11 37 16 64
+++ 4 15 47 66
Total 57 80 71 208
x² test, p =0.000
f
Liver cancer tissue
YAP O-GlcNAc Merged DAPI
YAP O-T241-YAP Merged DAPI
High
Low

Supplementary Figure 8. YAP is O-GlcNAcylated at Thr241 and O-GlcNAcylation of YAP is high in liver cancer tissues and established liver cancer cell lines.

(a) The YAP-FLAG (either WT or indicated mutant) expressing plasmids were transfected into Bel-7402 cells. Exogenous YAP-FLAG was immunoprecipitated by an anti-FLAG antibody. Co-immunoprecipitation of O-GlcNAc was analyzed using an anti-O-GlcNAc antibody (left panel). O-GlcNAclation of exogenous YAP-FLAG (either WT or indicated mutant) was measured via anti-TAMRA antibodies (upper) and HRP labeled-Streptavidin (lower) respectively in Bel-7402 cells by enzymatic labeling of O-GlcNAc sites experiments (right panel).

(b) Comparison of amino acids sequences around the O-GlcNAc site within YAP proteins among Homo sapiens (Thr241), Mus musculus, Xenopus laevis and Drosophila melanogaster.

(c) p-LATS1 and total-LATS1 in Bel-7402 and SMMC-7721 cells under treatment of DMSO or PuGNAc with GlcNA, as measured by Western blotting.

(d) O-GlcNAcylation of YAP in liver cancer tissues and established cell lines. Endogenous YAP was immunoprecipitated by an anti-YAP antibody in liver cancer and its paired adjacent normal liver tissue (upper and lower panels) as well as in indicated established liver cancer cell (i.e. SMMC-7721, Bel-7404, Bel-7402, Huh7, HepG2 and SK-Hep1) and hepatocyte (THLE-3 and HL-7702) lines (middle panel). Co-immunoprecipitation of O-GlcNAc was analyzed using an anti-O-GlcNAc antibody (upper and middle panels) and an anti-O-T241-YAP antibody (lower panel), respectively.

(e) TMA using anti-O-T241-YAP antibodies. Data from O-T241-YAP (Fig. S4q), total-YAP (Fig. 1a), global O-GlcNAcylation (O-GlcNAc, Fig. 1a), CTGF (Fig. 1a) and Ki67 (Fig. 1a) were analyzed by a chi-square test.

(f) Subcellular localization of YAP, O-GlcNAc and O-T241-YAP in liver cancer tissue, as measured by IF experiment. Scale bar, 500 μM. The representative IF images of liver cancer tissues from 12 independent patients are shown. High and low indicates YAP has relative higher or lower levels in different liver cancer tissues.

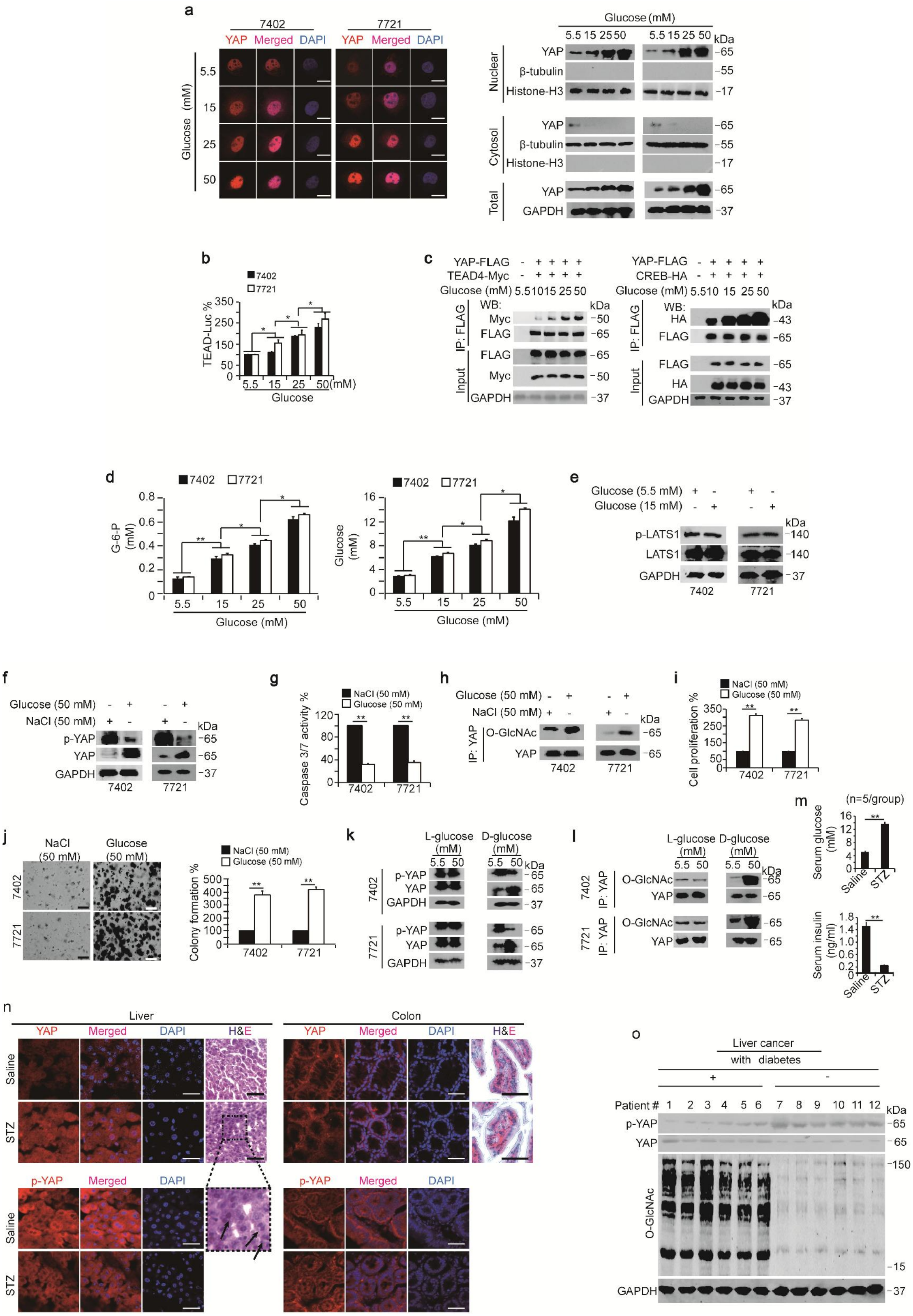
a
7402
7721
YAP Merged DAPI
Glucose (mM)
5.5
15
25
50
Glucose (mM)
5.5 15 25 50
kDa
Nuclear
Cytosol
Total
YAP
β-tubulin
Histone-H3
GAPDH
-65
-55
-17
-37
b
7402
7721
TEAD-Luc %
5.5 15 25 50(mM)
Glucose
c
YAP-FLAG
TEAD4-Myc
CREB-HA
Glucose (mM)
IP: FLAG
Input
WB:
Myc
FLAG
HA
GAPDH
-50
-43
d
G-6-P (mM)
Glucose (mM)
e
Glucose (5.5 mM)
Glucose (15 mM)
p-LATS1
LATS1
-140
f
Glucose (50 mM)
NaCl (50 mM)
p-YAP
g
Caspase 3/7 activity %
h
IP: YAP
O-GlcNAc
i
Cell proliferation %
j
NaCl (50 mM)
Glucose (50 mM)
Colony formation %
k
L-glucose (mM)
D-glucose (mM)
l
m
(n=5/group)
Serum glucose (mM)
Serum insulin (ng/ml)
Saline
STZ
n
Liver
Colon
YAP Merged DAPI H&E
p-YAP Merged DAPI
Saline
STZ
o
Liver cancer
with diabetes
+
-
Patient # 1 2 3 4 5 6 7 8 9 10 11 12
p-YAP
YAP
O-GlcNAc
GAPDH
-150
-15

Supplementary Figure 9. Glucose stimulates YAP.

(a) Glucose stimulated nuclear accumulation of YAP, as measured by a confocal microscopy assay. Bel-7402 and SMMC-7721 cells were treated with glucose at indicated concentrations for 24 h before harvest for examination. Scale bar, 20 μm (left). The nuclear and cytosol components of cells were prepared by a nuclear extract kit (Active motif, Carlsbad, CA, USA) in Bel-7402 cells treated with indicated increasing concentrations of glucose. The levels of YAP were subsequently examined by WB (right).

(b) Bel-7402 and SMMC-7721 cells harboring pUAS-LUC/TEAD-Gal4 system were cultured in media containing indicated concentrations of glucose for 24 h, and cellular luciferase activity was subsequently measured. The data from cells treated with 5.5 mM glucose are arbitrarily set to 100%.

(c) Glucose facilitated the binding between YAP and TEAD or CREB. Bel-7402 cells were transfected with YAP-FLAG and TEAD4-Myc or CREB-HA, and cultured in media containing indicated concentrations of glucose for 24 h. YAP-FLAG was immunoprecipitated by an anti-FLAG antibody, followed by Western blotting analysis of TEAD4-Myc or CREB-HA.

(d) Bel-7402 and SMMC-7721 cells were treated with indicated increasing concentrations of glucose. Cell lysates (1.25×10^6 cells/100 μl lysis buffer) were harvested and examined for intracellular G-6-P and glucose.

(e) p-LATS1 and total-LATS1 in Bel-7402 and SMMC-7721 cells under treatment of indicated concentration of glucose, as measured by Western blotting.

(f) Western blots of p-YAP and YAP in Bel-7402 and SMMC-7721 cells treated with 50 mM glucose or NaCl.

(g) Caspase activities were measured by a Caspase 3/7 Glo luciferase assay in Bel-7402 and SMMC-7721 cells treated with 50 mM glucose or NaCl. The data from cells treated with NaCl (50 mM) are arbitrarily set to 100%.

(h) O-GlcNAcylation of YAP. Bel-7402 and SMMC-7721 cells were treated with 50 mM glucose or NaCl. Endogenous YAP was immunoprecipitated by an anti-YAP antibody and Co-immunoprecipitation of O-GlcNAc was analyzed using an anti-O-GlcNAc antibody.

(i-j) Cell proliferation and colony formation capacities were measured by an MTT-based assay (i) and soft agar colony formation assay (j). The data from cells treated with NaCl (50 mM) are arbitrarily set to 100%.

(k) Western blots of p-YAP and YAP in Bel-7402 and SMMC-7721 cells treated with indicated

concentrations of L-glucose or D-glucose.

(l) O-GlcNAcylation of YAP in Bel-7402 and SMMC-7721 cells treated with indicated concentrations of L-glucose or D-glucose. Endogenous YAP was immuno-precipitated by anti-YAP antibodies and O-GlcNAcylation of YAP was detected by anti-O-GlcNAc antibodies.

(m) Serum-glucose and serum-insulin in mice treated with saline or STZ. N=5 per group.

(n) YAP and p-YAP were detected in the liver and colon from saline- and STZ-treated mice by confocal microscopic assay. The tissues were also stained by H&E. Arrows indicate dividing cells. Scale bar, 100 μm.

(o) p-YAP, total-YAP and global cellular O-GlcNAcylation in liver cancer tissues from patients complicated with diabetes or not.

Representative images are shown, and the data are expressed as the means + SD from three independent experiments. *, $p < 0.05$ and **, $p < 0.01$ indicate statistical significance. The data from Supplementary Fig. 9b and 9d were analyzed by a one-way ANOVA test, and the data from Supplementary Fig. 9g, 9i-9j and 9m were analyzed by a Student's t-test.

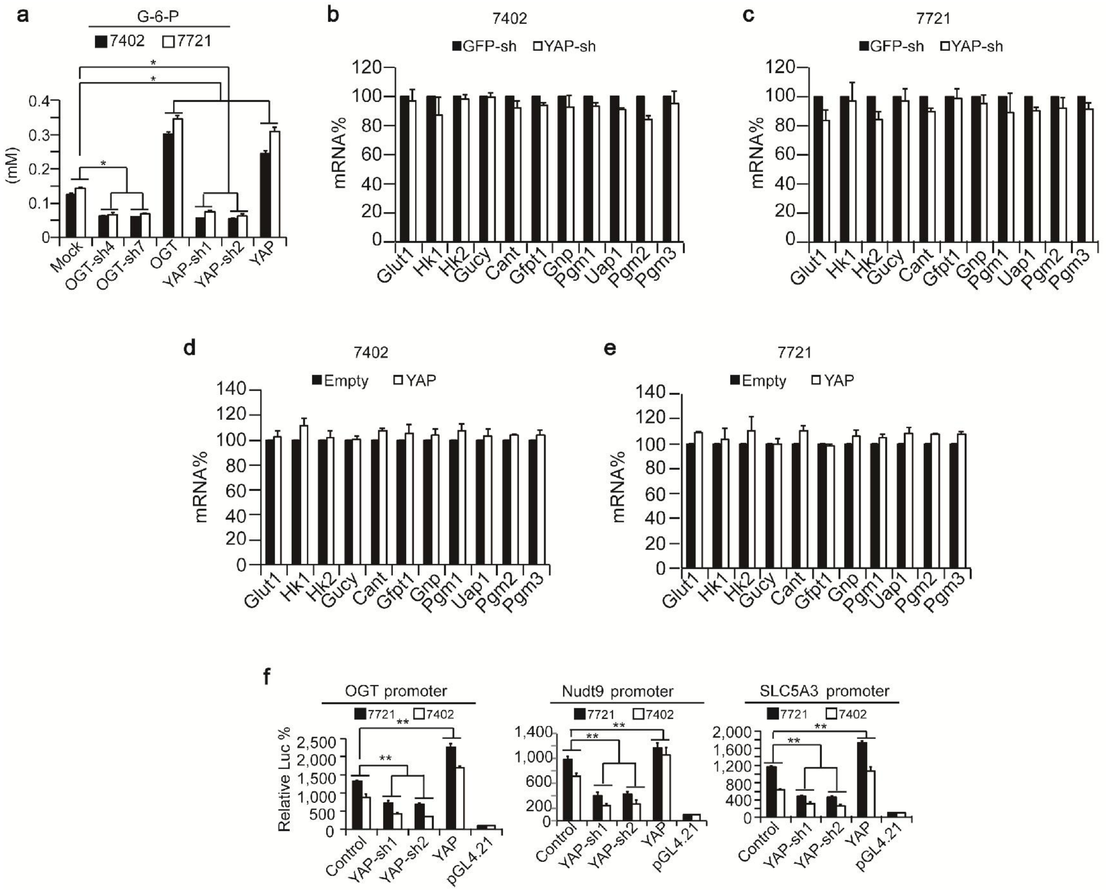
a
G-6-P
7402 7721
(mM)
Mock OGT-sh4 OGT-sh7 OGT YAP-sh1 YAP-sh2 YAP
b
7402
GFP-sh YAP-sh
mRNA%
c
7721
GFP-sh YAP-sh
mRNA%
Glut1 Hk1 Hk2 Gucy Cant Gfpt1 Gnp Pgm1 Uap1 Pgm2 Pgm3
d
7402
Empty YAP
mRNA%
e
7721
Empty YAP
mRNA%
f
OGT promoter
Nudt9 promoter
SLC5A3 promoter
7721 7402
Relative Luc %
Control YAP-sh1 YAP-sh2 YAP pGL4.21

Supplementary Figure 10. YAP increases intracellular G-6-P and transcription of HBP genes.

(a) YAP and OGT up-regulated intracellular G-6-P. Cell lysates (1.25×10^6 cells/100 μl lysis buffer) were harvested and intracellular G-6-P was examined by a kit from Sigma in control (Mock, without any treatment) and Bel-7402 or SMMC-7721 cells with OGT/YAP knocked down or overexpressed.

(b-c) The mRNA levels of genes as indicated in control and Bel-7402 cells (b) or SMMC-7721 cells (c) with YAP knocked down. The data from “GFP-sh” are arbitrarily set to 100%.

(d-e) The mRNA levels of genes as indicated in control and Bel-7402 cells (d) or SMMC-7721 cells (e) with YAP overexpressed. The data from “empty vectors” are arbitrarily set to 100%.

(f) Bel-7402 or SMMC-7721 cells were transfected with expressing plasmids as indicated. Promoter activities of *OGT*, *Nudt9* and *SLC5A3* genes were measured by a dual luciferase analysis. The data from pGL4.21 are arbitrarily set to 100%.

The data are shown as the means + SD from three independent experiments. *, $p < 0.05$ and **, $p < 0.01$ indicate statistical significance. The data from Supplementary Fig. 10a and 10f were analyzed by a one-way ANOVA test. The data from Supplementary Fig. 10b-e were analyzed by a Student’s t-test.

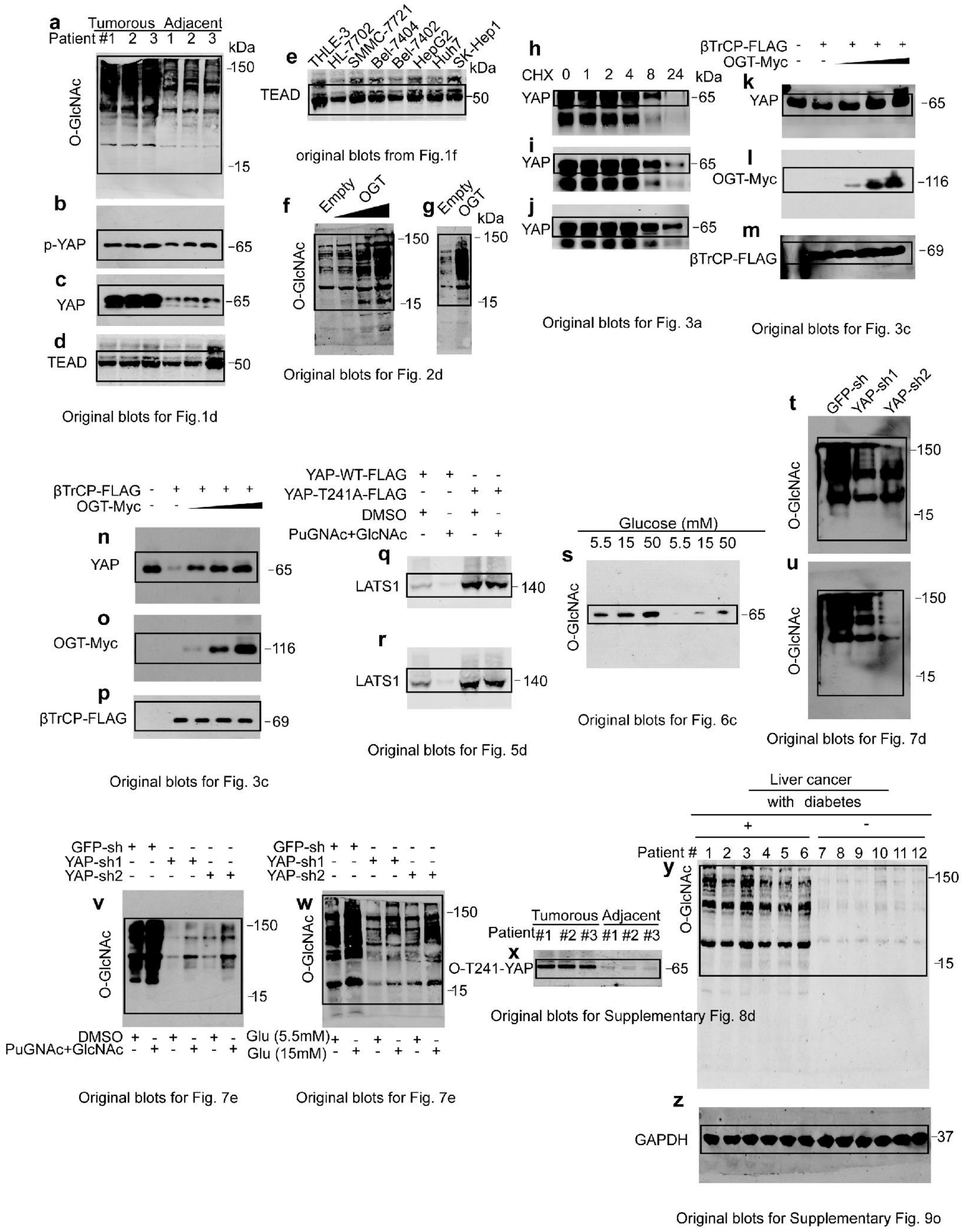
a
Tumorous Adjacent
Patient #1 2 3 1 2 3
kDa
150
15
O-GlcNAc
b
p-YAP
65
c
YAP
65
d
TEAD
50
Original blots for Fig.1d
e
THLE-3
HL-7702
SMMC-7721
Bel-7404
Bel-7402
HepG2
Huh7
SK-Hep1
kDa
TEAD
50
original blots from Fig.1f
f
Empty
OGT
O-GlcNAc
150
15
g
Empty
OGT
kDa
150
15
Original blots for Fig. 2d
h
CHX 0 1 2 4 8 24 kDa
YAP
65
i
YAP
65
j
YAP
65
Original blots for Fig. 3a
βTrCP-FLAG - + + + +
OGT-Myc - -
k
YAP
65
l
OGT-Myc
116
m
βTrCP-FLAG
69
Original blots for Fig. 3c
βTrCP-FLAG - + + + +
OGT-Myc - -
n
YAP
65
o
OGT-Myc
116
p
βTrCP-FLAG
69
Original blots for Fig. 3c
YAP-WT-FLAG + + - -
YAP-T241A-FLAG - - + +
DMSO + - + -
PuGNAc+GlcNAc - + - +
q
LATS1
140
r
LATS1
140
Original blots for Fig. 5d
s
Glucose (mM)
5.5 15 50 5.5 15 50
O-GlcNAc
65
Original blots for Fig. 6c
t
GFP-sh
YAP-sh1
YAP-sh2
O-GlcNAc
150
15
u
O-GlcNAc
150
15
Original blots for Fig. 7d
GFP-sh + + - - - -
YAP-sh1 - - + + - -
YAP-sh2 - - - - + +
v
O-GlcNAc
150
15
DMSO + - + - + -
PuGNAc+GlcNAc - + - + - +
Original blots for Fig. 7e
GFP-sh + + - - - -
YAP-sh1 - - + + - -
YAP-sh2 - - - - + +
w
O-GlcNAc
150
15
Glu (5.5mM) + - + - + -
Glu (15mM) - + - + - +
Original blots for Fig. 7e
Tumorous Adjacent
Patient #1 #2 #3 #1 #2 #3
x
O-T241-YAP
65
Original blots for Supplementary Fig. 8d
Liver cancer
with diabetes
+
-
Patient # 1 2 3 4 5 6 7 8 9 10 11 12
y
O-GlcNAc
150
15
z
GAPDH
37
Original blots for Supplementary Fig. 9o

Supplementary Figure 11. Represented images of original blots

(a-d) Western blots of O-GlcNAc (a), p-YAP (b), YAP (c) and TEAD (d) in liver cancer and adjacent normal liver tissues. The O-GlcNAc (a), p-YAP (b), YAP (c) and TEAD (d) blots in the box rectangles were presented in Fig. 1d.

(e) TEAD expression established hepatocyte (THLE-3 and HL-7702) and the indicated liver cancer cell lines. The TEAD blot in the box rectangles was presented in Fig. 1e.

(f-g) Overexpression of OGT stimulated O-GlcNAc levels in Bel-7402 (transfected with 1-4 μg of OGT-expressing plasmids) and SMMC-7721 cells (transfected with 4 μg of OGT-expressing plasmids). The O-GlcNAc blots in the box rectangles were presented in Fig. 2d.

(h-j) O-GlcNAcylation increased YAP stability. Protein synthesis was blocked by treatment with CHX (50 μg/ml) for the indicated times. The half-life of endogenous YAP in SMMC-7721 cells treated with DMSO (h) or PuGNAc (25 μM, i) with or without GlcNAc (4 mM, j) for 24 h. The YAP blots in the box rectangles were presented in Fig. 3a.

(k-m) OGT reversed βTrCP-induced YAP degradation in Bel-7402 cells. YAP (k), OGT-Myc (l) and βTrCP-Flag (m) expressions wer examined with βTrCP overexpressed in the presence or absence of transfection with an increasing amount of OGT-Myc plasmid (transfected with 1–3 μg of OGT-Myc-expressing plasmids). The YAP (k), OGT-Myc (l) and βTrCP-Flag (m) blots in the box rectangles were presented in Fig. 3c.

(n-p) OGT reversed βTrCP-induced YAP degradation in SMMC-7721 cells. YAP (n), OGT-Myc (o) and βTrCP-Flag (p) expressions wer examined with βTrCP overexpressed in the presence or absence of transfection with an increasing amount of OGT-Myc plasmid (transfected with 1–3 μg of OGT-Myc-expressing plasmids). The YAP (n), OGT-Myc (o) and βTrCP-Flag (p) blots in the box rectangles were presented in Fig. 3c.

(q-r) Mutation of Thr241 induced LATS1-YAP binding under stimulation of O-GlcNAcylation by combined treatment of GlcNAc (4 mM) and PuGNAc (25 μM). Expression plasmids for YAP-FLAG, WT or T241A were transfected into Bel-7402 and SMMC-7721 cells, and cells were treated as indicated for 24 h before analysis. YAP-FLAG was immunoprecipitated with anti-FLAG antibodies, and the association with LATS1 was measured in Bel-7402 (q) and SMMC-7721 cells (r). The LATS1 blots in the box rectangles were presented in Fig. 5d.

(s) Glucose stimulated O-GlcNAcylation of YAP at Thr241. Bel-7402 cells were transfected with WT and T241A YAP-FLAG, and cells were treated with glucose at the indicated final concentration for 24 h. Exogenous WT or T241A YAP-FLAG was immunoprecipitated with

anti-FLAG antibodies, and O-GlcNAcylation was measured by western blot using an anti-O-GlcNAc antibody. The O-GlcNAc blot in the box rectangles was presented in Fig. 6c.

(t-u) YAP stimulated O-GlcNAcylation. O-GlcNAcylation was analyzed by WB in Bel-7402 (t) or SMMC-7721 cells (u) with YAP knocked down. The O-GlcNAc blots in the box rectangles were presented in Fig. 7d.

(v-w) Stimulation of O-GlcNAcylation was dependent on YAP. Bel-7402 cells with YAP knocked down were treated with DMSO, PuGNAc (to a final concentration of 25 μM) with GlcNAc (to a final concentration of 4 mM) for 24 h before being harvested to test the O-GlcNAc level using WB (v). O-GlcNAc level was also tested by WB in Bel-7402 cells with YAP knocked down after treatment with the indicated concentration of glucose for 24 h (w). The O-GlcNAc blots in the box rectangles were presented in Fig. 7e.

(x) O-GlcNAcylation of YAP Thr241 in liver cancer and adjacent normal liver tissues. Endogenous YAP was immunoprecipitated by an anti-YAP antibody in liver cancer and its paired adjacent normal liver tissue. Co-immunoprecipitation of O-GlcNAc was analyzed using an anti-O-T241-YAP antibody. The O-T241-YAP blot in the box rectangles was presented in Supplementary Fig. 8d.

(y-z) O-GlcNAc levels in liver cancer tissues from patients complicated with diabetes or not were detected using WB. The O-GlcNAc (y) and GAPDH (z) blots in the box rectangles were presented in Supplementary Fig. 9o.

Supplementary Table 1. Intracellular metabolites that regulated by YAP in Bel-7402 and SMMC-7721 cells.

	Bel-7402				SMMC-7721			
		YAP				YAP		
Metabolites	Mock	O/V	sh1	sh2	Mock	O/V	sh1	sh2
Lactic acid (μM)	75.50±3.38	29.33±1.39 *	128.57±0.60 *	110.32±1.19 *	56.61±2.68	16.25±1.59 *	97.33±0.50 *	83.15±0.69 *
Glutamine (mM)	11.93±0.23	22.47±0.95 *	4.78±0.61 **	6.10±0.89 *	8.96±1.83	16.87±0.70 *	3.60±0.26 **	4.15±0.05 *
Glutamic acid (mM)	2.19±0.04	1.30±0.03 *	3.03±0.02 **	3.19±0.02 **	1.55±0.05	1.07±0.07 *	2.36±0.11 *	2.38±0.02 *
Acetyl-CoA (μM)	2.06±0.02	6.21±0.02 **	0.49±0.02 **	0.51±0.01 **	1.39±0.02	4.25±0.01 **	0.29±0.01 **	0.33±0.02 **
Fructose-6-P (μM)	5.96±0.45	14.80±0.35**	1.99±0.03 *	1.89±0.07 *	4.06±0.37	10.00±0.35 *	1.20±0.05 *	1.31±0.13 *
Glycogen (mg/l)	35.90±1.47	33.01±3.71	32.46±2.63	36.51±0.77	23.50±0.20	21.85±1.08	19.99±0.15	19.88±1.55

*,p<0.05, and **, p<0.01 versus Mock as analyzed by a one-way ANOVA test. Intracellular metabolites were measured in Bel-7402 and SMMC-7721 cells with or without YAP overexpressed (O/V) or knocked down by sh1/2. The data indicated the concentration of metabolites of cell lysates generated by adding 100 μl lysis buffer into 1.25×10^{6} cells.
